# Supportive care or exhausted neglect: the role of microglia at the end stage of prion disease

**DOI:** 10.1172/JCI186940

**Published:** 2024-12-02

**Authors:** Victoria A. Lawson

**Affiliations:** Department of Microbiology and Immunology, The Peter Doherty Institute for Infection and Immunity, The University of Melbourne, Victoria, Australia.

## Abstract

The transmissible nature of prion diseases enables reproduction of neurodegeneration in small animal models that faithfully follows the disease process observed in the natural disease of animals and humans. This allows the temporal development of disease to be investigated and correlated with pathology in a complex brain environment. In this issue of the *JCI*, Makarava et al. describe a shift in microglia morphology from an active phagocytic phenotype to a passive association with neuronal cell bodies. Whether this morphological change reflects a supportive action of microglia in response to neuronal impairment or exhaustion of PrP^Sc^-laden microglia remains to be determined. However, if microglial populations effectively contain PrP^Sc^ propagation early in the infection process, as the current study suggests, identifying ways to maintain or enhance the function of this cell population could be the key to prolonging patient survival.

## Prion diseases

Prion diseases are invariably fatal neurodegenerative diseases associated with the accumulation of a misfolded form of the normal cellular prion protein. While the function of the normal cellular form of the prion protein (PrP^C^) is expansive, the misfolded form of the protein, termed PrP^Sc^, is pathognomonic of prion diseases and synonymous with the transmissible agent, or prion.

As with many other neurodegenerative disorders, including Alzheimer’s and Parkinson’s diseases, disease-associated protein misfolding can be sporadic, with no defined cause, or familial and associated with mutations in the prion protein gene. However, in prion diseases, the misfolded protein aggregates are also able to transmit the disease through template-directed misfolding of PrP^C^. Although template-directed misfolding of other neurodegeneration-associated proteins has been described as prion-like, the propagation of PrP^Sc^ misfolding can generate infectious prions. Inoculation of small animals with PrP^Sc^, therefore goes beyond modeling a disease that naturally occurs in a wide range of mammals, including humans, but fully reconstitutes it.

Following intracerebral inoculation of wild-type mice with prions, PrP^Sc^ can typically be first detected half-way through the incubation period in wild-type mice, with the other hallmarks of prion disease, vacuolation, and glia cell reactivity, specifically astrocytes, appearing shortly thereafter (1). Infectious titers of prions peak within the first third of the incubation period (2), which correlates with the appearance of toxic PrP species that affect neuronal function through changes in long-term potentiation ex vivo (3). However, synaptic loss and clinical signs are typically not observed until the final weeks of the infection period (typically 90% of the incubation period) and correlate with rapid disease progression. Despite this detailed characterization of the pathological and clinical progression of disease, it remains to be determined what precipitates the extraordinarily rapid clinical progression of prion disease.

In this issue of the *JCI*, Makarava et al. (4) used an intraperitoneal infection with the strain of prions designated as synthetic strain leading to overweight (SSLOW) to investigate the role of microglia in controlling or contributing to the rapid clinical progression that characterizes prion diseases. The authors’ observation of microglia enveloping but not engulfing neurons in late clinical stages of the disease is also observed in the brains of mice infected with ME7, RML, and 22L prion strains and in the brains of patients with Creutzfeldt-Jakob disease (CJD) at terminal disease. This observation attests to the widespread occurrence of this microglia phenotype in prion disease regardless of prion strain type or host species.

## Prion strains

Variation in the clinical presentation, incubation period, disease duration, and pattern of pathology observed in the brains of animals and humans affected by prion diseases has been attributed to strain variation, which, in the absence of a nucleic acid–encoded conventional infectious agent, is believed to be encoded by different conformations adopted by the misfolded prion protein (5). For research, prion strains, such as ME7, RML, and 22L, were developed by adaptation of sheep scrapie prions to rodents (mice or hamsters), where the subsequent disease has all the attributes of the disease observed in its natural host (6). Medically relevant human prion strains, including those from Gerstmann-Straussler-Scheinker syndrome (7) and CJD (8), have been adapted to mice.

The SSLOW prion strain used in Makarava et al. (4), however, wasn’t, adapted from a naturally occurring prion strain arising from a sheep scrapie or human prion disease. It was developed by passage of full-length recombinant PrP that had been converted into a cross-β-sheet amyloid conformation, then amplified in vitro using the brain homogenate of a Syrian hamster. Inoculation of SSLOW prions into Syrian hamsters has been previously shown to induce disease with all the hallmarks of a prion disease (9, 10). In many regards, SSLOW represents a prototypic prion disease while at the same time being completely synthetic in origin.

## Disease development in SSLOW-infected mice

**Figure 1:**
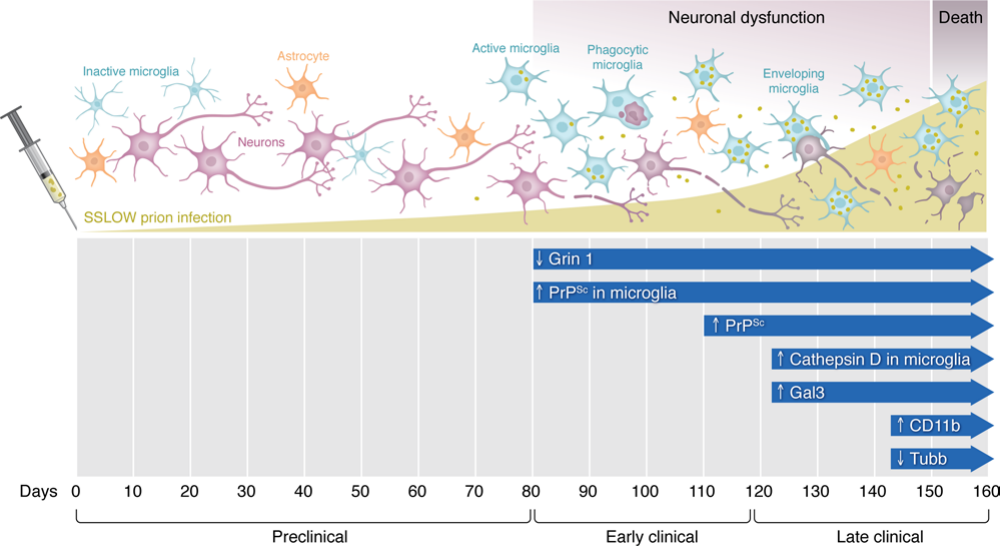
Clinical disease in wild-type mice infected with SSLOW prions coincides with microglia that have a neuron-enveloping but not engulfing phenotype. During the preclinical phase of SSLOW prion infection there is little evidence of neuronal dysfunction or microglial activation. Prions are likely to be propagating and have not reached detectable or clinically relevant levels. Early clinical disease is marked by neuronal dysfunction (indicated by decreased Grin 1 expression) and PrP^Sc^ accumulation in microglial cells. Microglial cells later envelop but do not appear to engulf dysfunctional neurons, despite expression of markers of microglial activation and phagocytic activity (indicated by cathepsin D, Gal3, and CD11b). There is a marked increase in the size of the microglial population; however, they do not appear to prevent PrP^Sc^ accumulation in the late clinical phase. At the terminal stage of disease, there is a decrease in the neuronal marker Tubb3, suggesting neuronal death. The effects on astrocytes remain unclear.

Makarava et al. (4) characterized disease progression in mice following an intraperitoneal inoculation of SSLOW prions (Figure 1). In this model, clinical onset occurred 120 days post infection (dpi) and terminal disease, as defined by weight loss, at approximately 160 dpi. The first half of the incubation period appeared relatively benign, without detectable levels of PrP^Sc^ or altered markers of neuronal function or microglial activation. PrP^Sc^ was first detected in the brain in association with microglia at around 80 dpi (50% incubation period), with neuronal envelopment observed at 100 dpi (65% of the incubation period), clinical onset and microglial activation at 120 dpi (75% of the incubation period), and overt neuronal loss, as assessed by decreased Tubb3 expression, detected in the last 15 days of the incubation period (or at 90% of the incubation period). Microglia that enveloped neurons appeared to have phagocytic capacity, as determined by the expression of cathepsin D, CD11b, and Gal3. However, there was limited evidence of complete neuronal engulfment, and disease progression was not CD11b dependent. At the terminal stage of disease, PrP^Sc^-laden microglial expressed early markers of apoptosis (i.e., caspase-3).

The observation of a unique morphology of microglia that appeared to envelop neurons without engulfing them was observed in the final phase of the infection period and coincident with clinical onset. This morphology was not restricted to SSLOW-infected mice and was observed in the brains of mice infected with ME7, RML, and 22L prion strains, and in the brains of patients with sporadic CJD at terminal disease. This latter observation would appear to negate the argument of incomplete neuron engulfment by microglia due to early euthanasia in animal models. Rather, this microglia phenotype could represent a supportive response to neuronal dysfunction, which was indicated by the downregulation of Grin1 expression. Alternatively, a failure of microglial function, perhaps reflected by caspase activation in microglia, could lead to the arrest of phagocytosis and the accumulation of PrP^Sc^ during the clinical phase of disease.

## Microglia

Microglia are a central protagonist in the pathogenesis of neurodegenerative diseases. They arise from yolk sac hematopoiesis and migrate to the brain to become a self-sustaining population following the closure of the blood-brain barrier (11). Microglia play a critical role in development and maintenance of synaptic plasticity and neurogenesis and in response to injury or infection develop a proinflammatory or antiinflammatory phenotype. Cytokines produced by proinflammatory microglia promote a proinflammatory astrocyte phenotype (12), which can drive pathology in neurodegenerative diseases and is detected in prion disease (13–15). In this regard, it will be of interest to characterize the response of astrocytes to the microglial-enveloped neurons described by Makarava et al. (4).

A recent study further described a small population of interferon-responsive microglia (IRM) that appear to envelop and engulf neurons during development and are also present in pathologies including viral infections and Alzheimer’s disease (16). Microglia in mice lacking the obligate IFN-I receptor IFNAR-1 (*Ifnar1^–/–^*) failed to phagocytose damaged neurons during development. Type I interferons have been detected in the terminal stages of prion disease (17). However, the role of the interferon response and interferon-responsive microglia in prion infection was variable with prion infection of *Ifnar1^–/–^* mice both modestly prolonging (17) and shortening (18) disease duration. Nonetheless, these studies highlight the potential for small populations of microglial cells to have a notable effect on disease phenotype (19) and support further nuanced study to dissect the role of microglia in neuronal survival and death within the complex environment of the brain and neurodegeneration.

## Future directions

In the context of prion diseases and the current study, characterization of the microglial subpopulations throughout the infection period will be key to understanding the pathogenesis of prion disease and informing our understanding of microglia in neurodegeneration. If microglial populations effectively contain PrP^Sc^ propagation early in the infection process, as the current study suggests, identifying ways to maintain or enhance the function of this cell population could be the key to prolonging patient survival. Despite the challenges of studying prion diseases, the veracity of the in vivo models established within the prion field and the SSLOW model described in the current study offer the best possible models for understanding neurodegeneration caused by prion and prion-like proteins.

## Version 1 12/02/2024

Electronic publication
